# Construction Notes

**Published:** 1896-11-28

**Authors:** 


### CONSTRUCTION NOTES.

**Glasgow Samaritan Hospital.**

This building, which has just been opened, is in two portions—the hospital and the administrative offices—which are connected by corridors five feet wide. The hospital block consists of two floors. On each floor there is one ward capable of accommodating ten patients, and a couple of wards have accommodation for two patients each. There are, besides, convalescent-room, ward scullery, linen-room, and complete sanitary accommodation. The principle of small wards has been kept in view in designing the hospital. The smaller wards will be utilised for the more serious cases; and the arrangements also permit of the larger wards being divided when necessary. The style of the bed used in the hospital merits more than a passing mention. It is fitted with movable side-bars and movable foot and bar. The arrangements and fittings of the wards have been devised on a wise and generous plan. The flooring here, as throughout the entire building, is oak-polished; and the walls, both at ceiling and floor, are rounded, so that there can be no corners where dust can accumulate. The heating is effected by hot-water pipes and also with open fires. The bath-room and lavatory accommodation is excellent. The administration block is three storeys in height. The ground floor contains board-room, waiting-room, matron's apartments, and surgeon's apartments. On the first floor there are bed-rooms for nurses, and also a nurses' sitting-room; while on the upper floor accommodation is provided for the servants. The kitchen is situated on the topmost floor. A hoist is provided for the operating-room. The pathological department, which is in one of the outbuildings, and the laundry are still incomplete in their appointments. The architects for this handsomely-appointed building are Messrs. M'Whannel and Rogerson, of West Regent Street, Glasgow, whose designs have been carried out at a cost of between £10,000 and £11,000.

**Passmore Edwards' Convalescent Home, Cranbrook, Kent.**

This home, situated on a beautiful and healthy site, is in connection with the Metropolitan Hospital, London, which some years ago was left a legacy of about £10,500 by the late Mr. Henry Spicer. The building provides accommodation for six men, six women, and six children, and the administrative staff, consisting of matron, nurses, and servants. In the centre of the building is the principal entrance, approached by a carriage drive. On the ground floor of the entrance hall is the matron's sitting-room and stores. The hall leads into a corridor, which connects the right and left wings of the building. On the right is the men's day-room with a small room adjoining, which can be used for a sick nurse or relapsed case if necessary. The lavatory on this floor is placed in a small block across the corridor, from which it is isolated. The block is cross-ventilated, and is shut off from the main building. Special attention has been given to the sanitary arrangements, which are of the latest and most approved patterns. The dining-room is situated centrally, and is common to men, women, and children. Behind the dining hall is the kitchen, back stairs, and usual offices, such as scullery, pantry, larder, laundry, &c. These are connected to the main building by a covered way. On the first floor, over the principal entrance, is the matron's bed-room, adjoining the men's dormitory, into which it has an inspection window. A nurse's bed-room is next to the women's ward. The matron and nurse have also their private bath-room, &c., between their bed-rooms. The patients' bath-rooms and lavatories are below, isolated, yet within easy reach of the dormitories, which are above the day-rooms below. The children's dormitory is over the dining-hall, with a bed-room for a nurse next to it. There is an inspection window in her bed-room looking into the children's room, so that at any hour they can receive attention. The back stairs lead up to the servants' bed-room. A male attendant has a bed-room, reached by a private staircase, over the outer offices. Externally, the building will be faced with good hard local red bricks. The effect of the whole is homely and simple in design. The site consists of three acres, well sheltered from the north by rising land at the back of the premises. When completed, the building, of which Mr. Charles Grieve was the architect, will cost about £3,000.

